# Mortality impact of implementing maternal perinatal deaths surveillance and response quality-of-care improvement strategy for maternal and newborn care, northeast Namibia: a quasi-experimental study

**DOI:** 10.7189/jogh.16.04050

**Published:** 2026-04-03

**Authors:** Gloria Mutimbwa Siseho, Thubelihle Mathole, Debra Jackson

**Affiliations:** University of the Western Cape, School of Public Health, Cape Town, South Africa

## Abstract

**Background:**

Northeast Namibia’s neonatal mortality rate is higher than the national level. From June 2019, Northeast Namibia regional hospital implemented maternal perinatal death surveillance and response system as a part of quality improvement strategy to address increasing newborn deaths. Yet, no documentation exists pre-and-post implementation of the system. This study therefore aims to assess and describes the before-and-after mortality impact of its implementation.

**Methods:**

A pre-post intervention study design was conducted. Quantitative routine facility data was collected for women who gave birth and their babies from January 2019–December 2021. Data was gathered from maternity, neonatal and paediatric wards by the hospital health information system officer. The excel for Mac version 28.0.1.1 was used to calculate the proportions, rates/ratios, and descriptive statistics applied. Analysis was done for maternal, perinatal and newborn deaths, number and mode of deliveries, and caesarean section rates.

**Results:**

Institutional mortality statistically reduced except for stillbirths. Neonatal mortality rate fell from 11.4 to 7.7 per 1000 live births (*P* = 0.035), perinatal mortality rate fell from 30.0 to 24.3 per 1000 total births (*P* = 0.049), while maternal mortality rate statistically reduced from 339.6 to 111.6 per 100 000 live births (*P* = 0.015). The still birth rate reduced marginally from 18.4 to 17.3 per 1000 total births, but was not statistically significant (*P* = 0.361). Average percentage intrapartum stillbirths reduced from 46.6 to 33.3%. The proportion of neonatal deaths in the first week of life increased from 75.4 to 83.6%.

**Conclusions:**

High perinatal (stillbirths and early neonatal deaths) accounted for most deaths within the neonatal period. Thus, improving the quality-of-care around childbirth and immediate postnatal care when most neonates are highly vulnerable requires urgent health system programmatic and policy attention. Overall, implementation of maternal perinatal death surveillance and response strategy proved feasible in improving care and reducing maternal and newborn mortality.

Globally, under-five deaths are declining faster than the neonatal mortality, with nearly half (47%) of the under-five deaths in the neonatal period [1,2]. There are above 2.6 million newborns and two million stillborn annually, many from preventable causes [1] resulting from poor quality-of-care rendered around childbirth and immediate postnatal care [1,3–6]. An estimated 50% of the stillbirths occur during intrapartum period [3], or results from complications during labour and childbirths [7]. The first week of life accounts for two million deaths, with a million on the day of birth [3,6]. Intrapartum stillbirth rates indicate the level of quality-of-care provided during labour [8]. While newborn deaths within the first 24 hours indicate the quality-of-care provided during labour and immediate postnatal care [8], making the time around childbirth the most vulnerable for child survival [1,9–17]. According to World Health Organization (WHO) estimates, 58% of neonatal deaths and half of maternal deaths can be prevented by providing quality of care during labour and childbirth [3, 18].

Poor quality-of-care accounts for 61% of neonatal deaths and half of maternal deaths [3], and is now the biggest barrier to reducing mortality than insufficient access [19]. Investing in quality-of-care around time of birth, especially care for the sick and small newborns, could save three million babies and women each year [20].

Maternal perinatal death surveillance and response (MPDSR) is a detailed qualitative analysis of the reasons and situations surrounding maternal and perinatal deaths [21]. It is a vital segment of the quality-of-care improvement efforts to lessen maternal deaths, as well as modifiable stillbirths and neonatal deaths [21]. The MPDSR can reduce mortality, however, its implementation is often suboptimal, especially in low- and middle-income countries [22].

Globally there has been an increase in deliveries occurring in health facilities, making it easier to collect information on facility deaths [9]. The WHO recommended maternal death reviews in 2004 [23], and maternal death surveillance review (MDSR) in 2013 [24]. Perinatal deaths reviews were included to expand to MPDSR in 2016 [9,22]. The MPDSR intervention is used by the facility deaths review committee to analyse events surrounding a death, recommend, implement, monitor, and evaluate implemented interventions [21]. While in 2021, WHO and United Nations International Children’s Emergency Fund (UNICEF) published implementation materials, also referred to as tools or implementation roadmap guiding countries on how to implement MPDSR [21]. Also, in 2021, the WHO guidance on consideration for synergy and alignment in implementing MPDSR as part of maternal and newborn quality-of-care efforts was published [18].

This study, conducted at an intermediate referral hospital in Rundu, northeast Namibia, reveals increasing neonatal and infant mortality rates: from 21 to 27 and 49 to 62 per 1000 live births, respectively, between 2007–2013 [25]. Despite high antenatal care (96.3%) and skilled birth attendance (75%), only 47.7% of mothers and 3% of newborns received postnatal care within two days after birth [25]. However, facility deliveries rose significantly from 8399 in 2016 to 11 967 in 2020 [26–28], indicating improved maternal health care access.

In Namibia, until this study, though a national maternal perinatal and stillbirths’ deaths review committee existed, its functionality was weak [29]. Prior to this study the national deaths review guidelines existed with practical implementation biased towards maternal deaths review [28]. For example, during 2019, national death review committee only reviewed maternal deaths despite reports of stillbirths and newborn deaths that were reported at facility levels [30]. This was despite the availability of guidelines forms that made provision for perinatal deaths reviews [31]. Also, before to this study, the national guidelines missed maternal perinatal deaths review guiding principles, how to conduct systematic reviews and formulation of smart-measurable-attainable-realistic-and-time-bound recommendations [31].

The national maternal, perinatal and neonatal death review committee – confidential enquiry report, April 2018–March 2021, had maternal deaths only reviewed, and no perinatal deaths [30]. Confirming, how perinatal deaths surveillance and response within the MDSR prior to this study was neglected. Also, the figures contained in the report are only extracts from the health information system. This is despite the mention in the title of the report and provisions made in the national death review guidelines.

In Kavango Hospital, during an assessment at baseline we noted that though the facility had a minute book for recording meetings discussions, no recommendations were recorded; and MDSR meetings were held irregularly, *e.g.* after 3–5 months. In addition, prior to the first MPDSR skills workshop, the facility team (including beyond the study site) were not trained for the past 12 months and more. The team composition was large, 12–15 people, with no indicators monitored. Prior to this study, the few deaths reviews conducted were biased towards maternal deaths [32]. Perinatal and neonatal deaths reviews were neglected or almost not conducted, despite the provision in the national deaths review guidelines [28,29].

On contrary, prior to this study, no maternal and newborn quality improvement interventions including policy documents existed [33–35]. To address the high neonatal mortality, this study aims to assess the impact on maternal and newborn mortality before-and-after implementing the MPDSR Quality Improvement (MPDSR-QI) system, in northeast Namibia.

## METHODS

### Aim

This study aims to assess the impact on maternal and newborn mortality pre-and-post implementing MPDSR-QI system or interventions, northeast Namibia.

### Study design

A quasi-experimental study design was used to describe maternal, perinatal, and newborn mortality before-and-after strengthening implementation of the MPDSR-QI intervention. To determine the quality of services available and provided to pregnant women, mothers, and their babies, we conducted a quantitative analysis of the MPDSR-QI monitored routine facility data. The data was extracted from the health information system for January 2019–December 2021. The health information system officer collects data from different sources in the hospital and enters it into the district health information system computer or database.

### Study setting

As reported in our past paper [27], The Kavango East is located 730 km away from the capital city or two national referral hospitals. The region also has four district hospitals, 24 clinics and three health centres. According to the Namibia District Health Information System 2 data, the region’s hospital deliveries are increasing since 2016 with an average annual increase of above 700 deliveries between 2016–2020. For example, deliveries were 8823 and 11 967 in 2019 and 2020 respectively. Kavango East, accounts for 50.14% (n = 124 286) of the total population [36]. During 2013, 3% of newborns received postnatal care within two days after delivery [25], and less than half in 2019 and 2022 [26,27].

### Sampling

The northeast regional referral hospital was purposively sampled for several reasons. Among them, at the time of data collection it accounted for around half of the region’s facility births. Across the country, it is amongst the four hospitals with high volume of patients. It is a teaching facility and the only northeast regional referral hospital covering three districts’ hospitals, and Zambezi region, 500 km, far-northeast of the country. Due to the region’s poor perinatal and childhood indicators, it among the regions supported by UNICEF Namibia. Thus, UNICEF resources were used to support interventions to address quality-of-care around childbirth, of which MPDSR is part.

### Description of materials

We extracted and analysed routine data from the health information system using the MPDSR excel performance monitoring tool. The tool has an automated function to calculate percentages, rates, and ratios. For this study, we analyse only 14 indicators monitored by the MPDSR-QI committee. The indicators include institutional stillbirth rate (iSBR), intrapartum still birth rate (SBR), antepartum SBR, % intrapartum SBR, institutional early neonatal mortality rate, institutional perinatal mortality rate (iPMR), institutional neonatal mortality rate (iNMR), institutional maternal mortality rate, caesarean section rate, percentage of assisted deliveries, institutional low birth weight rate, institutional premature birth rate, percentage of neonatal deaths in first week and maternal deaths. The tool, also, has an element that captures and tracks recommendations and their implementation status. The implementation status has three coding categories:

1) the recommendation is still within the implementation timeline

2) the recommendation has passed the recommended implementation time frame

3) the recommendation has been completed.

### Data collection

Using the MPDSR tool this study reviewed data collected from various sources in the hospital on women who gave birth, and their babies delivered January 2019–December 2021. The sources included: maternity and neonatal units, outpatient department and paediatric ward. Home deliveries and perinatal deaths not registered in the hospital records through the health information systems by time of data collection were excluded. The hospital health information system officer collected and entered the data on total births, live births, stillbirths, newborn and maternal deaths. The year 2019 as the baseline, precedes the MPDSR-QI intervention, 2020–2021 represent the intervention period. The data for end-of-study was extracted in 2022. Across the three years (2019–2021) there was a continuous data collection, and monitoring of the deaths through the MPDSR-QI facility team. Gathering baseline data in 2019 faced challenges due to incomplete records, and nonfunctional death review committee. Poor record keeping is consistent with baseline results from the same site [26]. Follow-up sessions after trainings or field monitoring visits were also used as opportunity to collect data through identification of strengths, challenges, and lessons learnt.

To ensure quality assurance of routine data, health information system (MIS) and integrated disease surveillance officer was trained and mandated with MPDSR data too. The MIS officer’s role was 2-fold: to facilitate integration and sustained ownership of the MPDSR data within the ministry of health information system. Second, the experience of quality checks already exists, and can be applied to MPDSR data.

### Data analysis

Descriptive statistics were used to analyse data from the MPDSR excel monitoring tool. The analysis included calculations of proportions and rates into tables, figures, and text using the excel for Mac version 28.0.1.1. We calculated χ^2^ and *P*-values using Social Sciences Statistics (https://www.socscistatistics.com/tests/chisquare/default2.aspx). The chi square test is applied in this study among others because it tests for independence to determine whether two categorical valuables are related. We tested to see if there was an association in terms of proportions of monitored indicators and/or mortality before-and-after delivering MPDSR-QI interventions. The study assessed if providing a quality improvement strategy (before-and-after) is associated with mortality levels (increased or decrease or category one or two). The relationship of pre-and-post was assessed by applying a χ^2^ test. The analysis included assessing the capacity and functionality of the deaths review committee in formulating implementable and following-up on recommendations pre-and-post MPDSR skills building workshop.

Due to missing or incomplete documentation, this manuscript excludes analysis of the recommendations before this study, and during the pick of COVID-19.

Analysis of the health care providers and patients’ perception of the MPDSR-QI interventions is not included as it was beyond the study scope. However, our past paper [26,27] provides some insights particularly on women’s perception of quality-of-care they received.

## RESULTS

A total of 19 493 births records were reviewed, of those 19 119 were live births. Overall live births represented 98% of the total births (**Table 1**). Of the total births, 374 were stillbirths (fresh and macerated) and 210 were neonatal deaths (**Table 1**). During 2019–2022, MPDSR-QI committee monitored indicators proportions/rates/ratio varied. For example, on average maternal mortality ratio, institutional stillbirth rate and perinatal mortality rate was 183.1 per 100 000 live births, 17.4 and 28.2 per 1000 total births respectively (**Table 2**). On average, from 2019–2021 neonatal mortality of 11.0 per 1000 live births was recorded. The percentage for CSR, assisted delivery rate, antepartum SBR, and low birth weight stayed relatively stable.

**Table 1:** Total births, live births, neonatal deaths 2019 to 2021, intermediate hospital, northeast Namibia

Indicators	Baseline	Intervention	Intervention	Overall
	**2019**	**2020**	**2021**	**2019–2021**
Number of total births (denominator)	5526	7075	7292	19893
Number of total live births (denominator)	5008	6945	7166	19119
Number neonatal deaths, 0–28 d of life	57	98	55	210
Neonatal deaths rate (%) (per 1000 live births)	11.38	14.11	7.68	10.98
Number newborn Deaths 1–7 d	43	74	46	163
Newborn deaths rate (%) 1–7days	75.4	75.5	83.6	77.6
Number newborn deaths 8–28 d	14	24	9	47
Newborn deaths rate (%), 8–28 d	24.6	24.5	16.4	22.4
Number stillbirths (FSB and MSB)	118	130	126	374
Stillbirths rate (%) (FSB and MSB) per 1000 total births	21.4	18.4	17.3	18.8
Number antepartum stillbirths	62	82	84	228
Antepartum stillbirths rate (%) out of the total stillbirth births	52.5	63.1	66.7	61.0

**Table 2:** Summary maternal and perinatal data (2019–2021)

Indicator	Baseline	Intervention	Intervention	Overall	*P*-value
	**2019**	**2020**	**2021**	**2019–2021**	**2019 *vs*. 2021**
Number of births (denominator)	5126	7075	7292	19 493	
Number of live births (denominator)	5006	6945	7166	191	
Maternal deaths	17	10	8	35	
Maternal mortality ratio (per 100 000)	339.6	144.0	111.6	183.1	0.015
Perinatal deaths	154	219	177	550	
Perinatal mortality rate (%)	30.0	31.0	24.3	28.2	0.049
Stillbirths	118	130	126	374	
Stillbirth rate (%)	23.0	18.4	17.3	19.2	0.361
Neonatal deaths	57	98	55	210	
Neonatal mortality rate (%)	11.4	14.1	7.7	10.9	0.035
Caesarean sections delivery	74	87	110	271	
Caesarean section rate (%)	17.5	15.6	17.9	17	0.768
Assisted deliveries	45	72	54	171	
Assisted delivery rate (%)	0.9	1.1	0.8	0.9	0.463
Low birth weight	42	129	86	257	
Low Birth Rate (%)	10.8	10.8	11.6	11.1	0.507

### Institutional maternal mortality

Across the indicators, average institutional maternal mortality ratio was 183.1 per 100 000 live births. The institutional maternal mortality rate, fell from 339.6 in 2019 to 144.0 and 111.6 in 2020 and 2021 resulting in a statistically reduced mortality (*P* = 0.015).

### Institutional neonatal mortality

**Figure 1:**
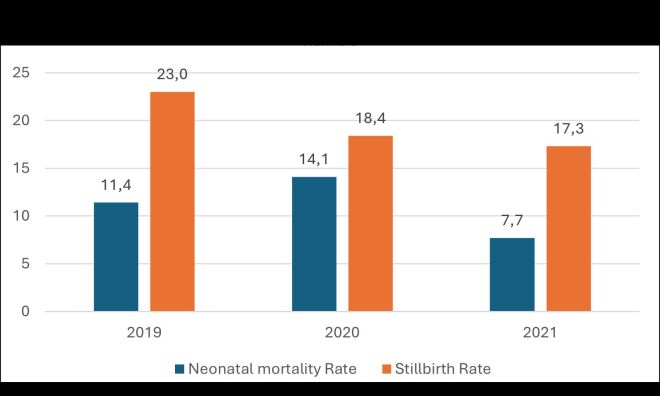
Neonatal mortality and stillbirth rate, 2019–2021, Intermediate hospital Northeast Namibia.

**Figure 2:**
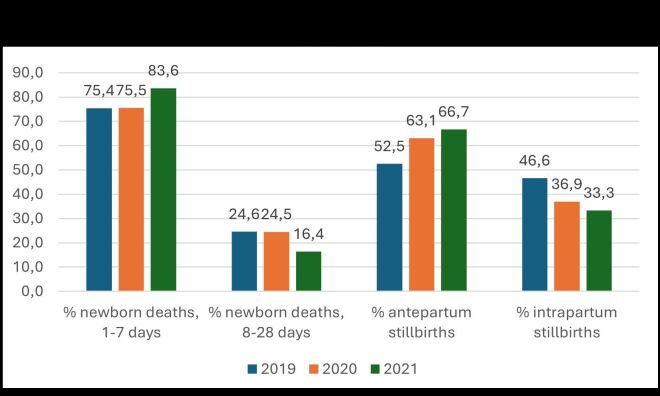
Perinatal and newborn deaths from routine data, 2019–2021, Intermediate hospital Northeast Namibia.

During the first year of implementing MPDSR-QI intervention, institutional neonatal mortality rate increased from 11.4 in 2019 to 14.1 per 1000 live births by 2020 (**Figure 1**). There was, however, a reduction in deaths from 11.2 at baseline to 7.7 end-of-study. The decline was statistically significant (*P* = 0.035). On contrary, there was an increase in early neonatal deaths (1–7 days) when comparing with deaths within 8–28 days (**Figure 2**).

### Institutional stillbirths

The institutional stillbirth rates fell slightly from 18.9 to 17.1 and 16.7 from 2019 to 2020 and 2021 per 1000 total births respectively. Overall iSBR reduction did not reach a statistical significance (*P* = 0.361) when comparing baseline and postintervention rates. Meanwhile, within the stillbirth rates, antepartum stillbirths increased by 10.7% (from 55.7 to 66.4%) out of the total institutional stillbirths (**Figure 2**). There was an overall decrease for intrapartum stillbirths of a similar 10.7% (from 44.3 to 33.6%) (**Figure 2**).

### Institutional perinatal deaths, low birthweight, and caesarean section deliveries

Perinatal deaths are defined as deaths among early neonates (1–7days after delivery) and stillbirths at or after 28 weeks of gestation [21,32]. Overall, the iPMR recorded an average of 28.3 per 1000 total births and statistically reduced from 30.0 to 24.3 per 1000 total births (*P* = 0.049). While, institutional low birth weight rate increased from 10.8 to 11.6 and not statistically significant (*P* = 0.507). During 2019–2021, the caesarean section delivery was 17.5–17.9% (*P* = 0.768); and assisted deliveries from 0.8–0.9% (*P* = 0.463), neither change was statistically significant.

### Causes of perinatal deaths

We found inconsistence in the top causes of perinatal deaths pre-and-post MPDSR-QI interventions. According to WHO definition of preterm birth, of the total stillbirths, 28.6% were preterm related births delivered at 30 weeks and/or below 37 gestational weeks. During pre-intervention, preterm births accounted for 81.8% of the total deaths, and for the primary (83.3%) underlying causes of deaths during the first 24 hours. Also, preterm deaths accounted for most (85.7%) of the deaths within the first week of life with 64.2% perinatal deaths. While, post-interventions, stillborn were the most (70%), deaths within the first 24 hours (66.7%), with 33.3% reported within the first week of life. For the detailed causes of perinatal and early neonatal deaths, analysis of the MPDSR-QI notes/min will be conducted, which is outside scope of this paper.

### Status of MPDSR-QI programme recommendations

Before June 2019, the maternal death reviews were held irregularly [29]. At pre-intervention stage (2019), despite having retrieved the death reviews minute book, recommendations/actions or minutes of those reviews were not traced or recorded. In May 2019, the facility death review committee was trained on perinatal death surveillance and response. Therefore, from June 2019–June 2020, the committee made recommendations, implemented and followed on their progress at each meeting. While, post June 2020, COVID-19 regulations disrupted the frequency of meetings resulting in inconsistence in capturing meeting recommendations. This resulted in a relapse to the before strengthening the perinatal ‘P’ within the MDSR, where no actions/recommendation were captured during the meetings. For this study, the MPDSR-QI committee reviewed and made a total of 20 recommendations from June 2019–June 2020. This paper focuses on the 16 recommendations made during October 2019–June 2020. June–September 2019 implementation of the MPDSR-QI was a training phase and systems were not completely in place, so this period has been excluded. Of the 16 analysed recommendations, 75% (12 of 16) were related to neonatal deaths, and 25% (four of 16) for maternal deaths. The decision to review a record was based on the latest perinatal death by the time of the meeting. Since maternal deaths were fewer, all were reviewed. The assumption is that if a case is well reviewed, specific measurable attainable/achievable realistic and timebound recommendations implemented and sustained, it can avert similar preventable death(s). The formulated recommendations address 12.5% (two of 16) of delays in receiving care within the health facility and 6.25% (one of 16) on standardisation of psychosocial support counselling for all women with a stillbirth delivery. While 18.75% (three of 16) of recommendations related to orientation on protocols and skills building on neonatal resuscitation. Another 18.75% (three of 16) were on strengthening health education to mothers including information on dangers of using traditional medicine on newborn babies and importance of seeking medical care early. Most 37.5% (six of 16) of the recommendations addressed issues related to poor record keeping and labour monitoring (use of partograph). Another 6.25% (one of 16) were on implementing best practice that saves lives in an emergency. For example, the team performed a perimortem caesarean section on a collapsed pregnant woman and saved two lives of newborns. A total of 81.25% (13 of 16) of the recommendations were health facility/health system related, fewer 18.75% relating to client/individual factors. Of the total recommendations made, 75% (12 of 16) were completed.

## DISCUSSION

### Summary of key maternal and newborn care

To the best of our knowledge, this is the first study in Namibia to document the impact of implementing a MPDSR-QI intervention for maternal and newborn care. During this study, it was for the first time the facility maternal deaths review committee were formally trained to include and learn how to conduct systemic surveillance and review. The study is among the few [8,26,27,30] that identified noteworthy quality-of-care strengths and gaps around childbirth and within the immediate postnatal period. In this study, implementing the MPDSR-QI intervention(s) demonstrated feasibility in reducing maternal mortality, iPMR and NMR and was statistically significant. This result is consistent with several authors [3,9,37] who argue that proving quality-of-care during labour, childbirth and immediate postnatal care period decreases mortality [3,9]. On contrary, despite these decreases, newborn deaths within 24 hours and early neonatal deaths were high. Below we discuss in detail the results.

### The contribution of the study to new knowledge

• The implementation of MPDSR-QI contributed significantly to enhancing perinatal death reviews within quality improvement frameworks.

• Advocacy efforts ensured integration into existing structures, while the Ministry of Health led nationwide design, coordination, and monitoring of interventions.

• Following the MPDSR skills workshop, national death review guidelines were updated to align with WHO/UNICEF principles, promoting standardised reviews.

• Hybrid follow-up sessions translated training into practice, enhancing quality of care, data management, and surveillance. Additionally, merging MPDSR and QI teams proved vital for overall intervention success, aligning with findings from a Uganda study [38].

### Quality-of-care impact on institutional maternal mortality

Maternal mortality is often described as the ‘litmus test’ of the health system, a measure of the system’s capacity to respond to women’s needs, especially during and after pregnancy and birth [23]. In this study, during the MPDSR-QI intervention phase, institutional maternal mortality rate statistically reduced by 33% (from 17 to eight deaths) from 2019–2021 (*P* = 0.015). The reduction is consistent with other studies [22,39] where maternal and neonatal mortalities reduced post implementing quality-of-care initiatives, and where, implementing MPDSR can reduce preventable deaths.

### Quality-of-care impact on stillbirths

Stillbirths is a sensitive marker of the health system strengths and a measure of quality-of-care provision in pregnancy and childbirth [40]. Most stillbirths, and almost all intrapartum stillbirths are preventable with high quality evidence-based interventions delivered before and during births [3,41]. Yet in this study, post MPDSR-QI interventions, very little (2.2%) decrease in overall iSBR was recorded from 18.9 to 16.7% per 1000 total births; the reduction level was not statistically significant (*P* = 0.361). Within the iSBR, antepartum stillbirths can be modifiable through the provision of high quality antenatal care [3]. Yet, in this study, most stillbirths occurred during antepartum period, before babies could take their first breaths. Stillbirths are preventable by providing high-quality evidence-based care before and during childbirth [3,9]. Suggesting that in this study, the level of quality-of-care delivered during labour and childbirth a time when most babies and mothers are highly vulnerable [10–12], was poor [8].

The study did not examine the causes of high antepartum stillbirths, as it was beyond its scope. However, the increase in antepartum stillbirths from 55.7 to 66.4% (2019–2021) suggests that maternal care during pregnancy may have been inadequate. The perinatal deaths surveillance and response review (PDSR) workshops equipped health care providers with essential knowledge and skills, which may have led to more accurate reporting of antepartum and intrapartum stillbirths. Additionally, quality improvement initiatives likely enhanced documentation and surveillance of maternal and perinatal deaths. The rise in hospital deliveries could also explain the increased antepartum stillbirth rates, as better recording practices facilitated the detection of cases, reflecting improved quality of care rather than a failure of quality improvement initiatives. Initial months of MPDSR-QI showed a spike in recorded deaths [42], attributed to enhanced record-keeping.

An estimated half of stillbirths occur during intrapartum, *i.e*. after labour has started but before delivery [3]. Almost all intrapartum stillbirths are preventable with high-quality evidence-based interventions delivered before and during labour/births [3, 41]. Intrapartum stillbirths further validate the degree of quality-of-care provided during labour [8]. In this study, intrapartum stillbirth decreased by 11% between 2019–2021. The decreases suggest that implemented MPDSR-QI interventions improved case management around labour and childbirth, and that reviewed recommendations fed directly into QI activities. This result is consistent with several authors [21,30,39] who reports similar findings. The 34% intrapartum stillbirth for this study is, however, high for an upper middle income country facility. Confirming that, unfortunately, the facility birth does not necessarily guarantee perinatal survival if the services are suboptimal [30].

### Quality-of-care impact on neonatal mortality

Neonatal mortality rate is the number of live born infant per year dying during 28 completed days of age [32]. Neonatal deaths refers to death 1–28 days, of which day one (first 24 hours of life); early (1–7 days of life); late (8–28 days of life) [32]. Most newborns die during childbirth or first 24 hours of life [16,17]. Neonatal deaths during the first 24 hours validates quality-of-care provided during labour, and immediate postnatal care [8,16,17]. Yet, in this study, neonatal deaths within 24 hours (66.7%) were high. Suggesting that poor quality-of-care is responsible for nearly 70% of the babies lost on their day of birth and immediate postnatal care period [8]. This result is high and inconsistent with the Bangladesh study of four districts (46.1%) combined [43]. Postnatal care is an opportune time to render care that prevents maternal and newborn deaths [37]. Yet, in this study, most newborns died during early postnatal period, which is on the same day of birth and first week of life. Validating the level of quality-of-care provided during labour and immediate postnatal care [8,16,17]. This result confirms the high vulnerability of neonates during childbirth and the immediate postnatal care period [10–12]. On contrary, decreases in NMR (*P* = 0.035) and iPMR (*P* = 0.049) post implementing MPDSR-QI approach achieved a statistically significant level.

Yet, despite the overall neonatal mortality rate reduction, newborn deaths in the first week of life was high (83.6%). This result is consistent with a Bangladesh study [43], where a similar, 83.6% early neonatal deaths was reported [43]. Our other papers from the same research site found poor postnatal newborn care immediately after birth [26,27]. The poor providers’ knowledge and skills, provider-client interactions in the management of maternal and newborn care complications, could answer to increases in early neonatal deaths [26]. Overall decreases in neonatal mortality rate could be due to implemented MPDSR-QI interventions around childbirth and early postnatal care period. However, despite high early neonatal deaths (1–7 days), overall perinatal deaths significantly reduced (*P* = 0.049).

### MPDSR quality-of-care improvement including status of recommendations

This study highlights the underrepresentation of perinatal deaths in reviews and data collection, despite the high mortality rate. The MPDSR-QI system was implemented to address increasing perinatal deaths. The study found that nearly 80% of review committee recommendations focused on neonatal deaths. The MPDSR-QI system demonstrated its effectiveness in improving data quality and management, with notable improvements in active surveillance, reporting, and analysis of reviewed death cases. The system also equipped health care workers with skills to correctly classify stillbirths and neonatal deaths, leading to a shift in numbers. The study attributes the improved data management to the training provided to providers, supervisors, and management. The MPDSR-QI system's capacity to address preventable causes of deaths likely contributed to the decrease in neonatal deaths in 2021.

The implementation of the MPDSR system has the potential to reduce mortality [22], however, COVID-19 regulations hindered the frequency of MPDSR-QI committee meetings, leading to a lack of recommendations to mitigate modifiable deaths. The high stillbirth rate necessitates further investigation for reduction strategies. Additionally, use of traditional medicine found during reviews requires further investigation, while increased perinatal deaths demonstrates improved maternal and newborn surveillance [42].

The observed proportional decrease in stillbirths alongside an increase in neonatal deaths highlights health care workers' increased capacity to report neonatal deaths within a non-blame framework of the MPDSR-QI system. Previous studies, such as Kashililika et al. [31] demonstrated inconsistent MPDSR implementation due to inadequate training and irregular meetings. However, the MPDSR-QI system in this study has effectively identified modifiable factors to enhance care through actionable recommendations. Consistent with existing literature, this approach shows promise in reducing maternal and neonatal mortality [22]; emphasising the need for sustained and scaled-up implementation of MPDSR-QI to address preventable perinatal deaths.

### Health system policy implications

Quality improvement is a continuous process to meet and exceed set standards and requires long-term implementation to sustain gains within the health system. Implementation of maternal and newborn care quality measures around childbirth and postnatal period when most mothers and their babies are most vulnerable is crucial. Considering increasing health facilities deliveries, the health system needs to empower health care workers with knowledge and skills on various MPDSR-QI interventions if deaths arising from poor quality-of-care [3] are to reduce. The health system capacity package therefore needs to include trainings in interpersonal communication [26,27], on evidence-based interventions [4,32,44], and adequate essential supplies and equipment, particularly for small and sick newborns [44].

### Health systems sustainability

Institutionalisation in health care refers to the consistent application of interventions post-project implementation with minimal external influence [8], particularly from donors. This study, which initiated MPDSR-QI interventions over three years, parallels the findings of the five-year Waiswa study [45] focused on enhancing newborn care across six hospitals. Although this research emphasised one high-volume hospital, its effects extended to improving perinatal and newborn care across 35 hospitals, demonstrating health system ownership and sustainability three years post-implementation. Key factors for sustainability included early engagement with local leadership, alignment of death review guidelines with global standards, and effective utilisation of local resources and data systems.

### Health system structural challenges

This study highlights improvements in maternity care, particularly regarding sustainability and ownership; however, it also reveals significant structural challenges within the health system. While maternity records were revised to comply with the new WHO Labour Care Guide [26], their availability remains inconsistent, with frequent stockouts despite initial support from UNICEF for printing. Previous research indicated adequate availability of essential health care supplies and medications [26,27]. In contrast, the irregularity of refresher training for providers on managing maternal and newborn complications raises concerns that demand attention from the health system. Additionally, inadequate postnatal care can be attributed to a shortage of human resources, as midwives are often occupied with deliveries, leading to insufficient care for mothers and newborns immediately after birth. Without addressing these human resource gaps and other structural issues, the long-term sustainability of maternal and perinatal death surveillance and response (MPDSR-QI) interventions is at risk.

### Strength and limitations

This study is not without limitations. There was no comparison site, however, we used the before-and-after study design to measure performance. Strengths include that the reviewed data focuses on the facility MPDSR-QI monitored indicators and the results enables the description of maternal, perinatal and newborn mortality and outcomes relevant for the MPDSR-QI team for planning improvements. We include data on causes of deaths.

Use of routine data, which often has quality issues, is a limitation, however the intervention included data strengthening so this might be minimised. Another limitation was mixed reporting of routine data on normal *vs*. preterm births, non-segregation or lumping of number of deliveries by mothers *vs*. total births. The knowledge on perinatal concept note critical in calculations of ratios, rates and percent was low. These challenges were addressed through improvements that were done in MPDSR-QI, skills trainings, reinforcement of knowledge and skills through regular onsite follow up post trainings, and regular feedback on monthly reports. Formulated change ideas/recommendations during the interventions phase were critical in addressing routine data limitations.

There was potential Hawthorne effect as HCWs knew there was a study, however, the long term and embedded nature of the intervention (two years) might be expected to have reduced this effect. Another limitation is that the study does not measure longer term sustainability and results of the MPDSR-QI intervention. Another limitation was that COVID-19 pandemic regulations disrupted the frequency of meetings, resulting in poor implementation and documentation of recommendations. The disruption could have contributed to preventable deaths as modifiable actions which could have resulted from the facility meetings were not taken. The national level supported the facility teams through regular visits and helped them to develop change ideas that were used to address adverse outcomes. However, a strength of the intervention is the overall coordination, monitoring and leadership of national level quality assurance unit, including alignment of national death review guidelines with global guidance.

These results are informing ministry of health and social services with evidence-based intervention, *e.g*. the impact of implementing MPDSR-QI intervention for maternal and newborn. The scale up this intervention beyond the study site is a strength and a lesson learnt for similar settings. We recommend further investigation into causes of high stillbirths, including the pros-and-cons of traditional medicines usage on maternal perinatal and newborn outcomes.

## CONCLUSIONS

This study is the first in Namibia to document the impact of the MPDSR-QI strategy on maternal and newborn care. Results show its feasibility in improving care quality and reducing mortality in similar resource settings. The MPDSR-QI also enhanced maternal and newborn death surveillance and data management. High perinatal deaths highlight the need for improved childbirth and postnatal care, requiring urgent health policy attention. We recommend training for care providers in MPDSR-QI interventions, postnatal management, and communication skills. Increasing human resources for maternal and newborn care is essential for sustaining quality care toward universal health coverage.

Support from Namibia’s Ministry of Health and Social Services (MHSS) was crucial in the design and monitoring of MPDSR-QI interventions, which by end of study led to scaling up across 35 public hospitals. This study uniquely combines research advocacy and simultaneous intervention scaling, providing evidence for broader implementation.
